# Multi-Scale Spatial Concatenations of Local Features in Natural Scenes and Scene Classification

**DOI:** 10.1371/journal.pone.0076393

**Published:** 2013-09-30

**Authors:** Xiaoyuan Zhu, Zhiyong Yang

**Affiliations:** 1 Brain and Behavior Discovery Institute, Georgia Regents University, Augusta, Georgia, United States of America; 2 James and Jean Culver Vision Discovery Institute, Georgia Regents University, Augusta, Georgia, United States of America; 3 Department of Ophthalmology, Georgia Regents University, Augusta, Georgia, United States of America; National Institute of Mental Health, United States of America

## Abstract

How does the visual system encode natural scenes? What are the basic structures of natural scenes? In current models of scene perception, there are two broad feature representations, global and local representations. Both representations are useful and have some successes; however, many observations on human scene perception seem to point to an intermediate-level representation.

In this paper, we proposed natural scene structures, i.e., multi-scale spatial concatenations of local features, as an intermediate-level representation of natural scenes. To compile the natural scene structures, we first sampled a large number of multi-scale circular scene patches in a hexagonal configuration. We then performed independent component analysis on the patches and classified the independent components into a set of clusters using the K-means method. Finally, we obtained a set of natural scene structures, each of which is characterized by a set of dominant clusters of independent components.

We examined a range of statistics of the natural scene structures, compiled from two widely used datasets of natural scenes, and modeled their spatial arrangements at larger spatial scales using adjacency matrices. We found that the natural scene structures include a full range of concatenations of visual features in natural scenes, and can be used to encode spatial information at various scales. We then selected a set of natural scene structures with high information, and used the occurring frequencies and the eigenvalues of the adjacency matrices to classify scenes in the datasets. We found that the performance of this model is comparable to or better than the state-of-the-art models on the two datasets. These results suggest that the natural scene structures are a useful intermediate-level representation of visual scenes for our understanding of natural scene perception.

## Introduction

How does the visual system encode natural scenes? What are the basic structures of natural scenes and what are their statistics? These are important research topics in both human and computer vision [1]–[9]. We now know that humans can grasp the gist of complex natural scenes quickly and remember extraordinarily rich details in thousands of scenes viewed for a brief period [10]–[12]. These observations impose significant constraints on neural representations and computations underlying natural scene perception. In current models of scene perception such as scene classification, there are two broad feature representations, global representations and local representations. Global representations such as GIST [8] and CENTRIST [9] encode structures of whole scenes and leave out local visual features and their spatial relationships at various scales. Local representations such as SIFT [13] and SURF [14] encode statistics of local features such as luminance gradients. Although both representations are useful and have some successes, the above observations on human scene perception seem to point to a representation that lies in between local and global representations.

We recently developed methods to explore concatenations of visual features at intermediate-level spatial and temporal scales in natural scenes and their applications in natural visual tasks. We developed a model of probability distribution (PD) of natural scene patches and derived a measure of visual saliency [15], a model of natural object structures and object detection in natural scenes [16], and a model of natural action structures and action recognition [17]. By extending this line of work to natural scene perception, we proposed Natural Scene Structures (NSSs), i.e., multi-scale spatial concatenations of local features, as an intermediate-level representation of natural scenes. Thus, any natural scene and category can be represented by a set of NSSs and their spatial arrangements. These NSSs encompass all possible combinations of local visual features, which include smooth patterns of luminance, textures, edges, junctions, and any combinations of these four patterns of luminance. Thus, the NSSs proposed here are quite different from many other scene statistics, including the second-order statistics, the statistics of edges in two-dimensional natural scenes [18]-[20], the statistics of natural luminance patterns [21], and the statistics of distances and surfaces in three-dimensional natural scenes [22], [23].

To compile the NSSs from images of natural scenes, we first sampled a large number of circular patches in a hexagonal configuration at multiple spatial scales. Then, we performed Independent Component Analysis (ICA) [24] on the circular patches and classified the Independent Components (ICs) into clusters using the K-means method. Finally, we obtained a set of NSSs with each corresponding to a set of dominant clusters of ICs.

To use the NSSs for scene classification, we examined a range of statistics of the NSSs compiled from two widely used datasets of natural scenes and modeled the spatial arrangements of the NSSs at larger spatial scales using adjacency matrices. We then selected a set of NSSs with high information about scene identifies, and used the occurring frequencies and the eigenvalues of the adjacency matrices as inputs to a Support Vector Machine (SVM) to classify the scenes in the datasets. We found that the performance of this model is comparable to or better than the state-of-the-art models on the two datasets. These results suggest that the concept of natural scenes as concatenations of NSSs is a useful model for our understanding of natural scene perception. Finally, we discuss possible neural representations of the NSSs.

## Results

### Possible neural codes of natural scenes

**Figure 1:**
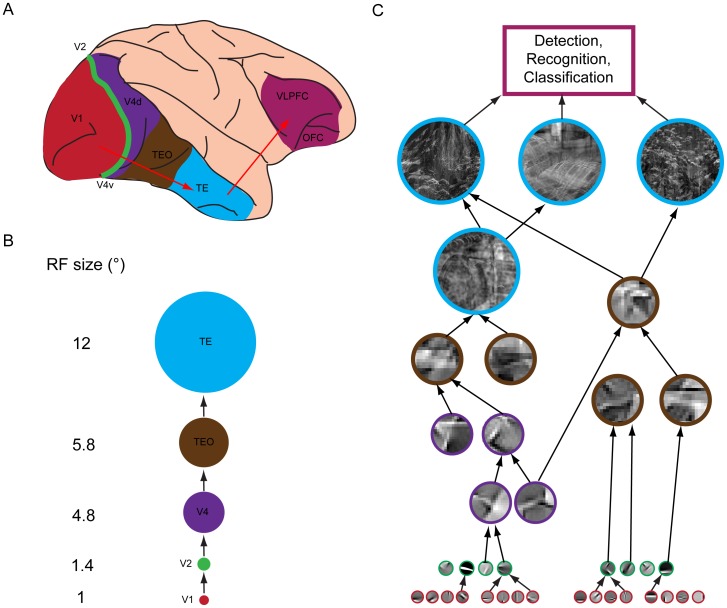
Hierarchical representations along the ventral visual pathway. (A), Information flow along the components of the ventral visual pathway of the macaque brain, including V1, V2, dorsal portion of V4 (V4d), ventral portion of V4 (V4v), the occipitotemporal cortex (TEO), and anterior part of the inferior temporal (IT) cortex (TE). The information along the ventral pathway is finally projected to the ventroloateral prefrontal cortex (VLPFC) and the orbitofrontal cortex (OFC) for tasks such as detection, recognition, and classification. (B), Receptive field (RF) sizes of the components along the ventral pathway for parafoveal vision. (C), Illustration of the representations along the ventral pathway. From V1 to TE, the encoded patterns become more and more complex. Based on the information encoded in a population of TE neurons, tasks (such as detection, recognition, and classification) are performed in the VLPFC and the OFC area. Adapted from [25]. Note that the basic oriented features are ICs of natural scenes. See descriptions in text.

Neural codes of natural visual scenes have been a focus of visual neuroscience in the last 50 years. Along the ventral visual pathway, neural codes of visual scenes become progressively complex from V1 to V2, V4, and the IT area (Figure 1). V1 neurons have a typical receptive field (RF) size of 0.1°–1° of visual angle in the central visual field, and encode a range of basic visual features such as orientation, contrast, and spatial frequency. The responses of V1 neurons can be described by the linear-nonlinear (LN) model [26]. Recent studies also showed that the complex cells in V1 have multiple excitatory and suppressive subunits, each of which is similar to an oriented bar [27]. V2 neurons have a typical RF size of 1.4°, integrate inputs from multiple V1 neurons, and respond selectively to both single and multiple orientations and shapes of intermediate complexity such as crosses and angles [28], [29]. V4 neurons have a typical RF size of 4.8°, integrate inputs from multiple V2 neurons, and respond selectively to curvature, orientation, and object-relative positions [30]. IT (including TEO and TE) neurons have a typical RF size of 5.8°–12° and respond selectively to complex configural relationships, shapes, and features (including skeletal shapes, faces, and places). The neural codes in the IT area are structural, configurational, and compositional and neural codes of populations of IT neurons are especially relevant for detection, recognition, and classification [31].

In summary, converging evidence suggests that neural codes of natural visual scenes are progressive concatenations of basic features (e.g., oriented bars) along the ventral visual pathway. Thus, we propose NSSs, i.e., topology-conserving, multi-size, multi-scale concatenations of visual features in natural scenes, as intermediate-level neural codes of visual scenes. In the next section, we briefly compare the NSSs to other models of visual codes.

### Relationship to other work

There are two lines of related work. The first line of work is computational models of scene classification. For this task, several low-level representations of visual features, including SIFT [13], SURF [14], and HOG [32], have been used. However, these low-level representations are limited since they are computed from small image patches and their spatial arrangements are usually ignored. To overcome these limitations, spatial pyramid matching [33], [34] and object bank representation [35] were developed, both of which can achieve good classification performance. In the spatial pyramid matching, images are partitioned into grids and concatenated histograms of low-level features in the grids are used for classification. In the object bank representation, scenes are represented by the responses of a set of object filters learned from training data. Another approach to scene classification is holistic representations where scenes are represented by global structures [8], [9] but the spatial arrangements of low-level or intermediate-level features are not explicitly examined. Our approach is different from these methods since the proposed NSSs provide a classification of scene patches of large sizes (∼3160 and ∼11620 pixels in two tested datasets) and encode local scaling-invariance, and the spatial arrangements of the NSSs in natural scenes are explicitly encoded by adjacency matrices (see below).

The second line of work is computational models of visual neurons. The forms learned from natural scenes in [36] are a set of shape features such as extended contours, multi-scale edges, textures, and texture boundaries. In [37], by extracting slowly varying signals from training data, the authors found some stimulus patterns that have features (e.g., non-orthogonal inhibition and side-inhibition) that resemble the response properties of some V1 complex cells. In [38], the authors trained a two-layer sparse deep belief network on natural scenes and obtained stimulus patterns (e.g., corners and junctions) that resemble the response properties of some V2 cells. In [39], the authors used a distribution coding model to learn correlational patterns (e.g., groups of oriented bars) in local image regions and found that the model can reproduce some response properties of V1 complex cells. The NSSs proposed here differ from these studies in several ways. First, each NSS is a concatenation of features (i.e., ICs) in 7 circular patches in a hexagonal configuration of multiple sizes and scales (see next section). Thus, the NSSs have much larger sizes than the stimulus patterns obtained by other models and each NSS has a range of natural variations. Second, the NSSs encompass all possible concatenations of local features in natural scenes, including smooth patterns of luminance, textures, edges, junctions, and any combinations of these four patterns of luminance. Thus, in principle, some NSSs have features to which V1 neurons respond selectively; some NSSs have features to which V2 neurons respond selectively; and some NSSs have features to which V4 or IT neurons respond selectively. Third, only three operations, i.e., categorization (via clustering), projection, and concatenation, are used to derive the NSSs. The ICs of circular scene patches are categorized into clusters, each of which shares similar orientations; each circular patch is projected to the clusters of the ICs; and the projected features in 7 circular patches in a hexagonal configuration of multiple sizes and scales are categorized as a set of NSSs (see next section). Finally, the spatial arrangements of the NSSs at various larger scales can be accessed. In graph theory [40], an adjacency matrix represents the connectivity of a graph and the eigenvalues of the adjacency matrix characterize the topological structure of a graph [41]. To apply this tool of graph theory, we partitioned scenes into grids and defined a neighboring relationship on the grids to obtain adjacency matrices. We then obtained the eigenvalues of the adjacency matrices as features for scene classification.

Finally, our approach can be contrasted with a computational model of rapid scene categorization that has some neurobiological basis [5]. In this model, a set of S- and C- units are trained to extract visual features at several levels. The basic S-units are Gabor functions and the S- and C- units at the higher levels are learned from the inputs from the lower levels via the tuning and max operations respectively. Our approach is different. First, the NSSs are compiled from natural scenes and there are no parameters of the NSSs to be learned (only the total numbers of the ICs, the clusters of the ICs, and the NSSs are determined via cross-validation). Second, the NSSs provide a classification of natural scene patches, and each has a PD. Third, the NSSs are very different from the optimal features of the S- and C- units because of the different operations in the two approaches. The NSSs are topology-conserving, multi-size, multi-scale concatenations of visual features in natural scenes, and any NSS looks like patches in real scenes. In contrast, the local topology and continuity in natural scenes are not conserved in the S- and C- units and thus the visual features of the S- and C- units are very different from patches in real scenes. Fourth, local scaling-invariance and scaling-variance are explicitly encoded in the NSSs since scene patches at multiple scales can be classified as a single NSS (scaling-invariant) or several NSSs (scaling-variant). Finally, the occurring frequencies of the NSSs and the eigenvalues of the adjacency matrices of the NSSs are features for scene classification.

In the following sections, we describe compiling the NSSs from datasets of natural scenes, concatenations of visual features in the NSSs, the spatial arrangements of the NSSs in natural scenes, the statistics of the NSSs, and scene classification using the NSSs and their spatial arrangements as features.

### Compiling natural scene structures

**Figure 2:**
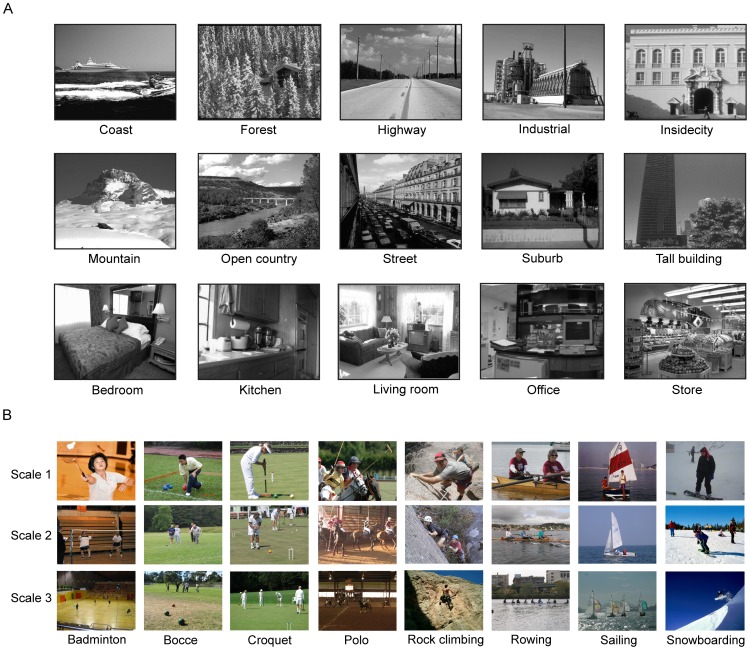
Scenes in the datasets of 15 scenes and 8 sports. (A), Sample images of the dataset of 15 scenes. There are 10 outdoor scenes and 5 indoor scenes. (B), Sample images of the dataset of 8 sports in three scales.

**Figure 3:**
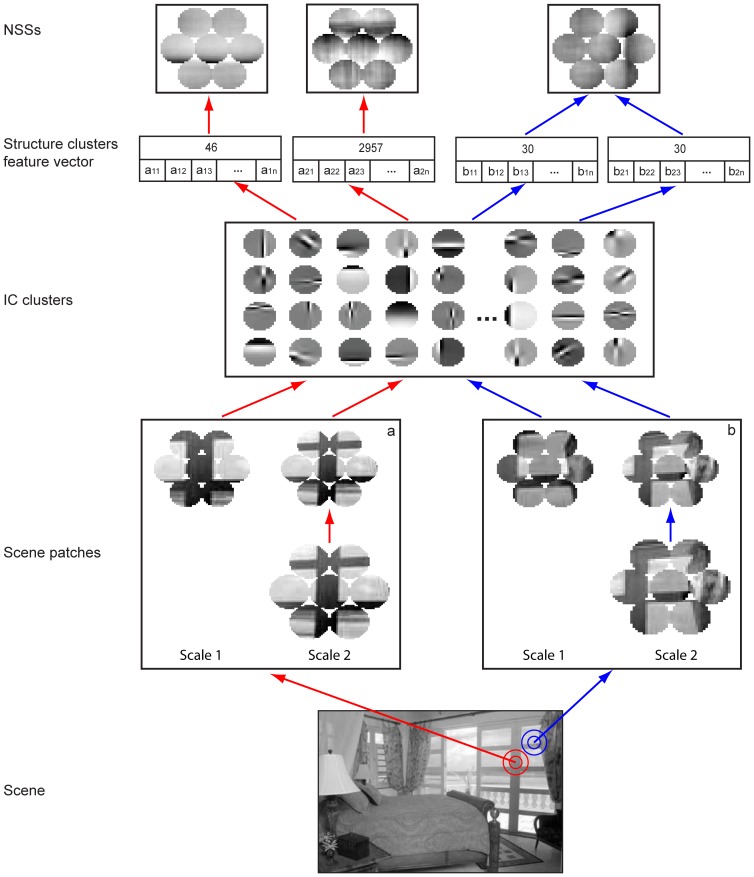
Procedure for compiling NSSs. First, we sampled circular scene patches in a hexagonal configuration at two spatial scales. Second, we performed ICA on the circular patches and classified the ICs into a set of clusters. For each IC cluster, we computed a feature, defined as the root mean square amplitudes of the ICs in the cluster, and obtained a feature space by merging the feature vectors of the circular patches in the hexagonal configuration. Finally, we digitized the feature space into a set of non-overlapping regions using the K-means method, assigned a structural index to each region, and designated all the patches in the hexagonal configuration that share the same structural index as a NSS. The patches at the hexagonal configuration at multiple sizes sampled at any location in a scene may be clustered to different NSSs (case a) or the same NSS (case b). Note that each NSS shown here is the average of all the patches that share the same structural index.

We propose NSSs as an intermediate-level representation of natural visual scenes. In this scheme, a visual scene is a spatial concatenation of a set of NSSs, each of which is a concatenation of a set of structured patches in natural scenes. Thus, any natural scene and category can be represented by a set of NSSs and their spatial arrangements. We took five steps to compile NSSs in two widely used datasets of natural scenes, a dataset of 15 scenes and a dataset of 8 sports (see Figure 2 and sec **Materials and Methods**). These steps are illustrated in Figure 3.

1. Sample a large number of circular patches in a hexagonal configuration at multiple spatial scales.
2. Perform ICA on all the circular patches *P* in the hexagonal configuration and obtain ICs at each spatial scale.
3. Fit Gabor functions to the ICs and classify the ICs at multiple spatial scales into a set of clusters (referred to as IC clusters) using the parameters of the fitted Gabor functions as features.
4. Project the circular patches to the IC clusters, compute the features of the circular patches, and pool the features of the patches in the hexagonal configuration at multiple spatial scales (Equation 1).
5. Partition the space of feature vectors into a set of NSSs.

There are several reasons for using ICs. First, the PDs of the amplitudes of ICs are statistically independent of each other. Second, the PDs of the amplitudes of the ICs of natural scenes are sparse. Third, ICs derived from natural scenes are much like the RFs of simple cells in V1. Finally, there is no need to set any parameters since ICs are learned from natural scenes. Also for these reasons, ICs are used instead of Gabor filters. Here, ICs are categorized according to orientations since neurons in V1, V2, and V4 are organized into orientation maps where neurons in a cortical column have similar tuning to orientations.

To compile the NSSs, we first sampled densely the images in the datasets as in other studies [42], [33]. At each selected location, we sampled seven non-overlapping circular patches in a hexagonal configuration. As shown in Figure 3, each circle is a circular patch and the configuration has multiple spatial scales. The diameters of patches at two spatial scales were 16 and 24 pixels, respectively. The rationale for using this configuration is to explore combinatorial concatenations of local visual structures at multiple spatial scales. To make computing more efficient, we down-sampled the patches at larger spatial scales. For the dataset of 15 scenes, we down-sampled the larger patches by 2/3 using bi-cubic interpolation. Thus, all the circular patches at the two spatial scales had the same diameter of 16 pixels.

**Figure 4:**
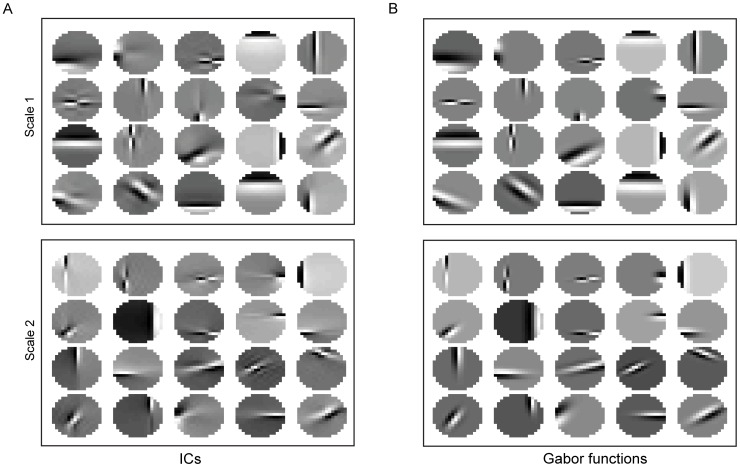
Fitting Gabor functions to ICs. (A), Examples of ICs of the image patches at two scales sampled from the dataset of 15 scenes. (B), Gabor functions that are fitted to the ICs shown in A.

We then performed ICA on the all the circular patches in the hexagonal configuration at the multiple spatial scales separately, and fitted Gabor functions to the obtained ICs. The fitting algorithm worked well, accounting for about 90% of the variance of the ICs. Figure 4 shows a few examples. To derive a compact representation of the ICs obtained at the multiple spatial scales, we performed clustering in the parameter space of the fitted Gabor functions. For this purpose, we used 6 parameters of the Gabor functions as in [17], i.e., 4 parameters of the Gaussian envelope and 2 parameters of the sinusoid carrier. Since different values of the parameters may correspond to the same Gabor function (e.g., adding 2π to the phase does not change Gabor function), we converted the estimated parameters to pre-set intervals (see **Materials and Methods**). Using these parameters, we clustered the ICs in two steps. First, we clustered the ICs into 16 equally divided orientations. Second, for each orientation, we performed the K-means clustering using the Euclidian distance in the parameter space of the Gabor functions as the metric.

Let *A* = {*A*_1_, *A*_2_, …, *A_m_*} denote the *i*-th IC cluster containing *m* filters at a spatial scale, each of which is a column vector with *l* elements, where *l* is the number of pixels in each circular patch. The feature, *a_i_*, of a circular patch *P* (which is a row vector with *l* elements) was calculated by projecting *P* to the *i*-th IC cluster as follows
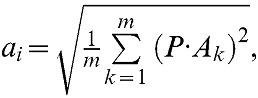
(1)


Thus, for *N* IC clusters, there are *N* features which form a feature vector for each circular patch. By pooling the circular patches in the hexagonal configuration, we obtained the 7×N features for the 7 patches in the hexagonal configuration at multiple spatial scales. Since the patches sampled from natural scenes do not uniformly pack the high-dimensional feature space, we partitioned the feature vectors into a set of clusters using the K-means method with the Euclidean distance metric. We call all the patches in the hexagonal configuration at the multiple spatial scales that fall in the same cluster a NSS. Since visual features at each scale are concatenated as a sample for categorization (via clustering), the patches at the hexagonal configuration at multiple scales sampled at any location in a scene may be clustered to different NSSs (case a) or the same NSS (case b) (Figure 3) which indicates local scaling-invariance. Thus, local scaling-invariance and scaling-variance are encoded in the NSSs. It is worth mentioning that for scene classification, we compiled NSSs from each scene category and pooled the NSSs from all the categories to form a master code book (see **Materials and Methods**).

In contrast to simple features such as ICs and SIFT descriptors, the NSSs are highly structured intermediate-level representations that are building blocks of natural scenes. Roughly speaking, as a result of the K-means clustering procedures, each of the NSSs contains a large set of patches of natural scenes that entails a specific pattern of spatial concatenation, ranging from simple to complex, of local features in natural scenes.

### Concatenations of visual features in NSSs

Since topology is conserved, i.e., the neighboring relationships among the pixels in the scene patches are maintained, the NSSs obtained here include all possible combinations of local visual features in small regions in natural scenes. The only limitations on the combinations are induced by the clustering procedures, which can be made looser or tighter depending on specific applications. Thus, the NSSs include smooth patterns of luminance, textures, edges, junctions, and any combinations of these four patterns of luminance and carry a variety of amount of information about natural scenes at multiple scales.

**Figure 5:**
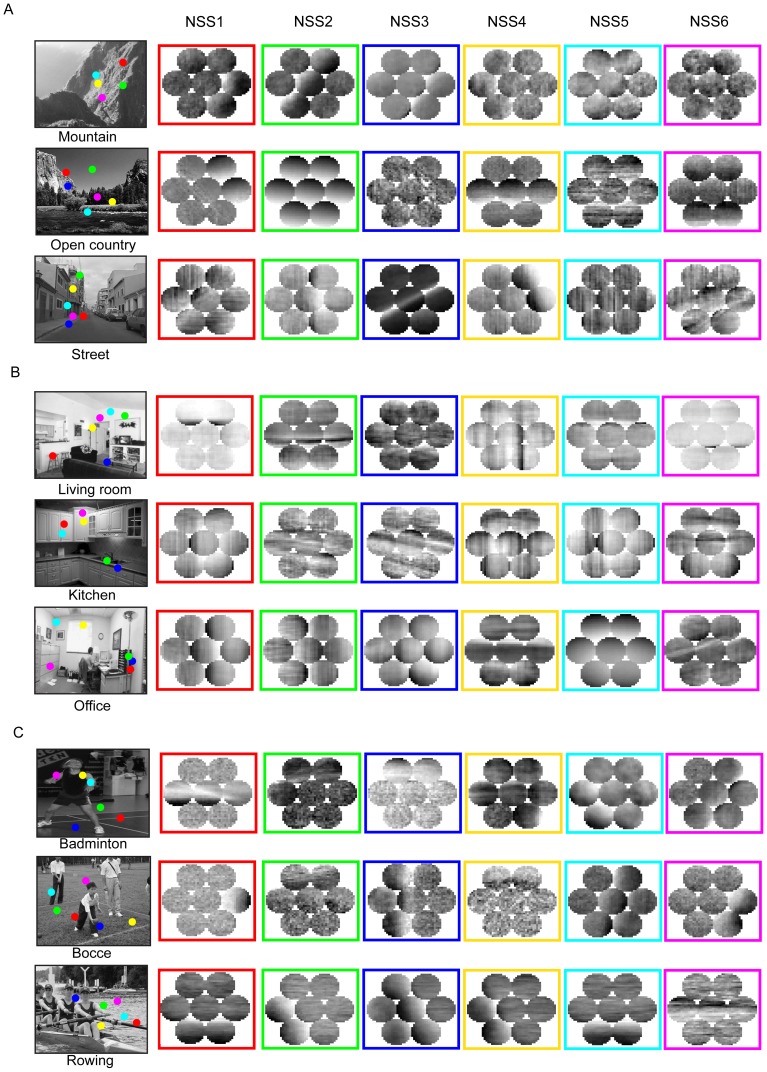
Examples of NSSs. (A), Six frequent NSSs compiled from each of the 3 selected outdoor scenes in the dataset of 15 scenes. The locations of the NSSs in the scenes and the boxes around the NSSs are indicated by the same color. (B), Same format as (A). Six frequent NSSs compiled from each of the 3 selected indoor scenes in the dataset of 15 scenes. (C), Same format as (A). Six frequent NSSs compiled from each of the 3 selected sports scenes in the dataset of 8 sports.

Figure 5 shows 6 frequent NSSs of each of the nine selected scenes categories in the datasets of 15 scenes and 8 sports. The NSSs shown here are actually the averages of the scene patches that share the same concatenations of local features (see above). We arranged the selected natural scenes in three groups, outdoor scenes (Figure 5A), indoor scenes (Figure 5B), and sports scenes (Figure 5C). The locations of the NSSs in the scenes and the boxes around the NSSs are indicated by the same color. The NSSs represent coarse but informative descriptions of a variety of scene components. For example, in the mountain scene (first row of Figure 5A), the first frequent NSS (indicated by red color) is a blurred texture pattern and is near the top of the mountain in the scene. In the open-country scene (second row of Figure 5A), the first frequent NSS is located at the boundary between the mountain and the sky and is a mixture of a blurred texture, a blurred edge, and smooth luminance ramps. In the living room scene (the first row of Figure 5B), the first frequent NSS is located at the chair and contains a sharp luminance change. In the kitchen scene, the first frequent NSS is located on the cabinet and contains a smooth luminance pattern generated by multiple light sources. In the bocce scene (the second row of Figure 5C), the first frequent NSS is located near the leg of the boy and is a mixture of a texture and a luminance jump. In the rowing scene (the third row of Figure 5C), the first frequent NSS is located at the boundary between the oar and the water surface and is a mixture of smooth luminance and a luminance jump.

**Figure 6:**
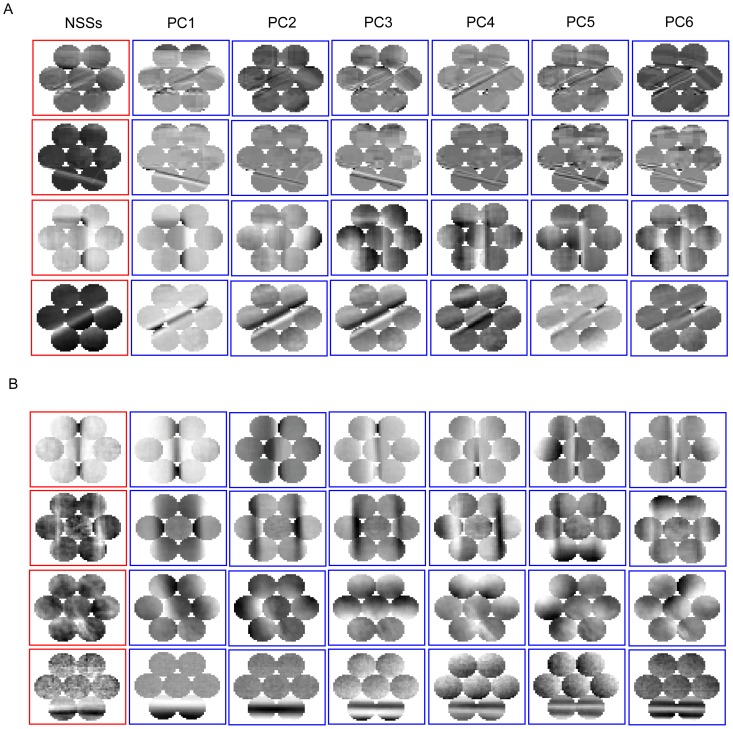
Variations in NSSs. (A), Four selected NSSs (marked with red boxes) and the top six Principal Components (PCs) (marked with blue boxes) for each NSS. The NSSs are selected from the dataset of 15 scenes. (B), Same format as (A). The NSSs are selected from the dataset of 8 sports.

Figure 5 shows the averages of the selected NSSs. In fact, each NSS contains a large number of scene patches that share a specific concatenation of local visual features, which means that each NSS has a range of natural variations. To examine variations in the NSSs, we performed Principal Component Analysis (PCA). Figure 6 shows the top six Principal Components (PCs) of each of the four NSSs selected from each dataset. Overall, the changes in luminance in the mean and the shown PCs are similar for each of the NSSs. As a quantitative measure, the first 150 PCs of the NSSs account for 34% more variance than those of natural scene patches. Thus, the NSSs are less variable than natural scene patches, as they should be.

### Spatial arrangements of NSSs in natural scenes

**Figure 7:**
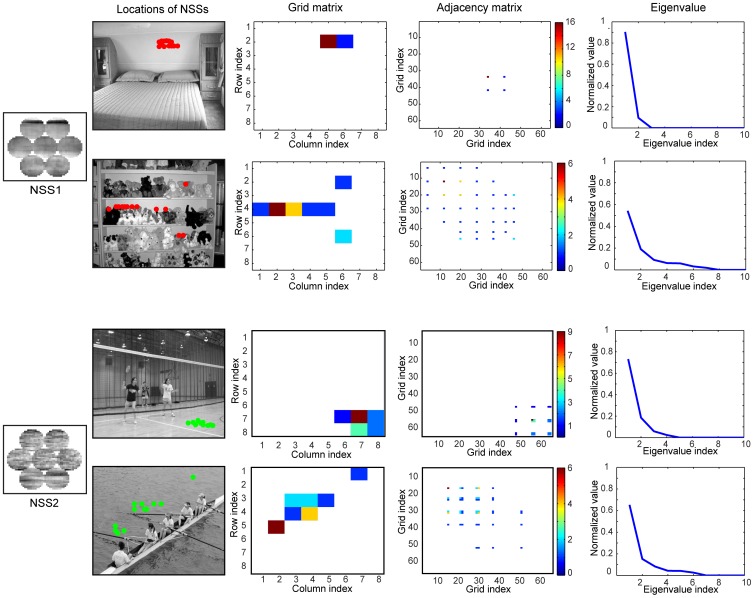
Spatial arrangements of NSSs. The grid matrices, the adjacency matrices, and the eigenvalues of the adjacency matrices for two selected NSSs in four selected images.



To model the spatial arrangements of individual NSSs, we first partitioned each scene into an 8×8 grid and obtained the occurring frequencies of individual NSSs within each grid location. Two examples are shown in Figure 7. We then used an adjacency matrix [40] to represent the neighboring relationship among the grid locations. To this end, we defined the distance *L* between two grid locations, *n*(*row_n_*, *col_n_*) and *m*(*row_m_*, *col_m_*) as

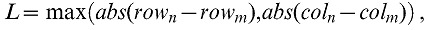
(2)where *abs*(·) denotes the absolute value function and *row* and *col* are the indices of the columns and rows of the grid. We assigned the minimal number of occurrences of the NSS at two grid locations to the corresponding element of the adjacency matrix if *L* was less than *L_c_* and the numbers of occurrences of the NSS at the two grid locations were greater than zero. The fourth column in Figure 7 shows 4 adjacency matrices (*L_c_* = 3). For the 8×8 grid, the adjacency matrix is a 64×64 symmetric matrix and most of its elements are zeros. Finally, we calculated the eigenvalues of the adjacency matrix, which are real numbers, and used the *N_c_* largest absolute eigenvalues to represent the spatial arrangement of the NSS. As shown in the fifth column of Figure 7, the eigenvalues of the adjacency matrices are more spread in scenes where the NSSs are dispersed across larger areas. To use this information for scene classification, we set *L_c_* and *N_c_* using cross-validation on the training datasets (see **Materials and Methods**).

**Figure 8:**
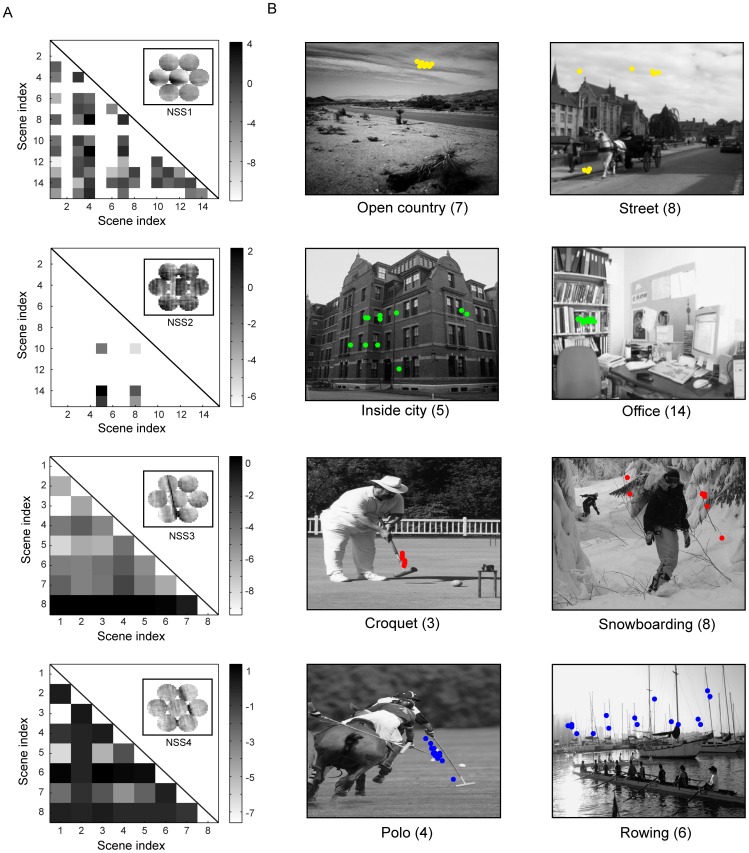
Discriminant information of NSSs. (A), The lower half of the Fisher discriminant matrix for four selected NSSs. The gray-scale bar indicates the logarithmic values in the base of 2. (B), Spatial locations of the NSSs in selected pairs of scenes. The numbers in the parentheses are the indices of the scene categories shown in Figure 2.

Using the top 2 to 5 eigenvalues of the adjacency matrices, we calculated the Fisher score (between-class variation divided by within-class variation) of individual NSSs for each pair of scene categories. Thus, we obtained a symmetric discriminant matrix for each NSS. The rows and columns of the discriminant matrix are the indices of the scene categories as shown in Figure 2. Figure 8 shows four Fisher discriminant matrices for four NSSs and four pairs of scenes. NSS1 and NSS2 were compiled from the dataset of 15 scenes and NSS3 and NSS4 from the dataset of 8 sports. Most of the scene pairs for which NSS1 has a high discriminant score include other scene categories vs. the highway scene (indexed by 3), the industry scene (4), the open-country scene (7), and the office scene (14). For example, the spatial arrangement of NSS1 is more spread in the street scene than in the open-country scene. Most of the scene pairs for which NSS2 has a high discriminant score include other scene categories vs. the inside-city scene and the street scene. For example, the spatial arrangement of NSS2 is more spread in the inside-city scene than in the office scene. Most of the scene pairs for which NSS3 has a high discriminant score include other scene categories vs. the snowboarding scene (8). For example, the spatial arrangement of NSS3 is more spread in the snowboarding scene than in the croquet scene (3). Most of the scene pairs for which NSS4 has a high discriminant score include other scene categories vs. the bocce scene (2), the rowing scene (6), and the snowboarding scene. For example, the spatial arrangement of NSS4 is more spread in the rowing scene than in the polo scene (4). Thus, the spatial arrangements of NSSs at larger scales can be indicative of scene categories even in cases where the numbers of occurrences of NSSs are not.

### Statistics of NSSs

**Figure 9:**
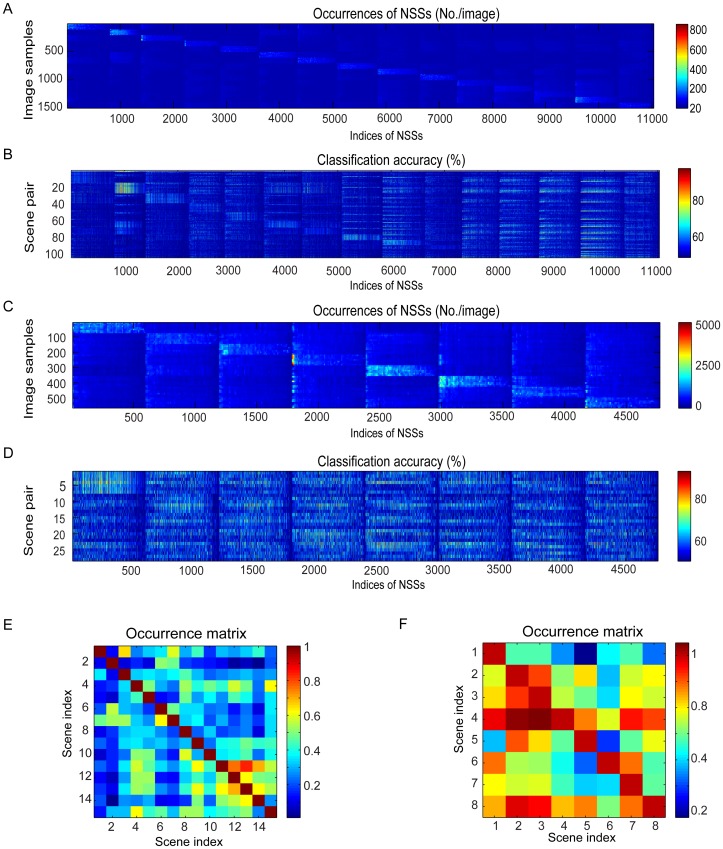
Statistics of NSSs. (A), Histograms of the NSSs in the training images. (B), Accuracy on classifying each pair of scene categories based on the occurring frequency and the spatial information of each NSS. (C, D), Same format as (A, B). (A, B) are the results for the dataset of 15 scenes. (C, D) are the results for the dataset of 8 sports. (E), Occurrence matrix for the dataset of 15 scenes. (F), Occurrence matrix for the dataset of 8 sports.

The master book has 11,028 NSSs for the dataset of 15 scenes and 4,761 NSSs for the dataset of 8 sports. Figure 9A shows the numbers of the occurrences of the 11,028 NSSs in the 1,500 images in the training set for the dataset of 15 scenes. The NSSs were arranged according to the indices of the scene categories from which they were compiled. Thus, most NSSs occur more frequently in the scene categories from which they were compiled.

To examine the information about natural scene categories carried by individual NSSs, we randomly separated the training set into two sub-sets (60% for training and 40% for evaluation) and used the occurring frequency and the spatial arrangement of each NSS to classify each pair of scene categories in the datasets. Figure 9B shows the matrix of classification accuracy on the dataset of 15 scenes. There are 105 pairs of scene categories in this database and the indices are arranged in the following way: the first 14 pairs are the first scene category vs. the rest of the categories in the order shown in Figure 2 and so on. Overall, the NSSs convey more information of the scene categories from which the NSSs were compiled.

We repeated the above procedures on the dataset of 8 sports. Figure 9C shows that most NSSs compiled from this dataset occur more frequently in the scene categories from which they were compiled. Figure 9D shows that the NSSs convey more information of the scene categories from which the NSSs were compiled.

Finally, we calculated a normalized occurrence matrix, M, to examine the overall occurrences of the NSSs in the two datasets. Each element *M_ij_* of the matrix is the total number of scenes of scene category *j* that contain any of the NSSs compiled from scene category *i* and each row of the matrix is normalized by the diagonal element of the matrix. Figure 9E shows the occurrence matrix for the dataset of 15 scenes. It is clear that the NSSs compiled from indoor scenes (index 11-15) also occur frequently in other indoor scenes. The occurrence matrix for the dataset of 8 sports is shown in Figure 9F. The NSSs compiled from bocce (2), croquet (3), polo (4), and snowboarding (8) also occur frequently in other scene categories. As it will become clear in the next section, these cross-occurrences affect scene classification adversely.

### Scene classification

To classify the scenes in the two datasets, we selected a set of NSSs that occurred in more than *M_c_* images in at least one of the scene categories, and obtained 11,028 and 4,761 NSSs for the dataset of 15 scenes and the dataset of 8 sports, respectively. We determined *M_c_* using a cross-validation procedure on a set of randomly selected training images (see **Materials and Methods**). We concatenated the occurring frequencies and the eigenvalues of the adjacency matrices of the selected NSSs as feature vectors, and fed them to an SVM to classify the scenes. SVM is suited for cases where feature vectors have many elements and a relatively small number of training samples are available [43]. The rationale was to let SVM make use of both types of the information to find the optimal decision boundaries between scene categories. SVM has been successfully used for object and scene classification [33], [44]. In this work, we used C-SVM with the 1-*χ* ^2^ kernel [45].
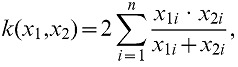
(3)


where *x*_1*i*_ is the *i*-th element of *x*_1_. For multi-category classification, we used the One-vs.-Rest strategy and the LIBSVM implementation of SVM [46], [47].

The results of our model and several other methods on the dataset of 15 scenes and the dataset of 8 sports are reported in Table 1 and Table 2, respectively. For the dataset of 15 scenes, we randomly selected 100 samples of each category for training and used the rest of the samples for testing. For the dataset of 8 sports, we randomly selected 70 and 60 samples of each category for training and testing respectively. The classification accuracies are the averages of the accuracies obtained in 5 training-testing runs. The classification accuracy of our model is 82.3% on the dataset of 15 scenes, which is the same as the state-of-the-art model (82.5%). The classification accuracy is 85.8% on the dataset of 8 sports, which is better than the best current model (84.4%).

**Table 1:** Performance of our model and other models on the dataset of 15 scenes.

Methods	15 Scenes
**Our model**	82.3±0.4%
**Niu et al. [48]**	82.5%
**Lazebnik et al. [33]**	81.4%
**Li et al. [35]**	80.9%
**Yang et al. [49]**	80.4%

**Table 2:** Performance of our model and other models on the dataset of 8 sports.

**Methods**	**8 Sports**
**Our model**	85.8±0.7%
**Dixit et al. ** [**50**]	84.4%
**Wu et al. ** [**51**]	84.2%
**Niu et al. ** [**48**]	78.0%
**Li et al. ** [**35**]	76.3%

To demonstrate the contributions of the components of our model to classification accuracy, we performed scene classification by dropping one or more components of the model. The results on the dataset of 8 sports (Table 3) show that classification performance was improved significantly (20% error reduction) for the concatenated features of the scene patches in the hexagonal configuration. The multi-scale coding strategy also achieved 10% error reduction relative to the best single-scale features on this dataset. The results also showed that adjacency matrices only improved classification slightly. This is presumably because the NSSs already encode considerable spatial information due to the dense sampling procedure and the large sizes of the circular patches and the hexagonal configurations relative to usual local visual features.

**Table 3:** Contributions of the components of our model to classification on the dataset of 8 sports.

**Methods**	**Without adj. matrices**	**With adj. matrices**
**Multi-scale & hexagonal config.**	85.1±0.5%	85.8±0.7%
**Best single-scale & hexagonal config.**	83.4±0.3%	84.2±0.3%
**Best single-scale & circular patch**	79.4±0.6%	80.0±0.8%

Since the dataset of 8 sports has considerable scale variations (Figure 2), we extracted patches at four spatial scales. The high accuracy of our model indicates that the NSSs proposed here can encode multi-scale information effectively. Unfortunately, we could not include more spatial scales in our model for the dataset of 15 scenes since the image resolution (∼300×250 pixels) is too low.

**Figure 10:**
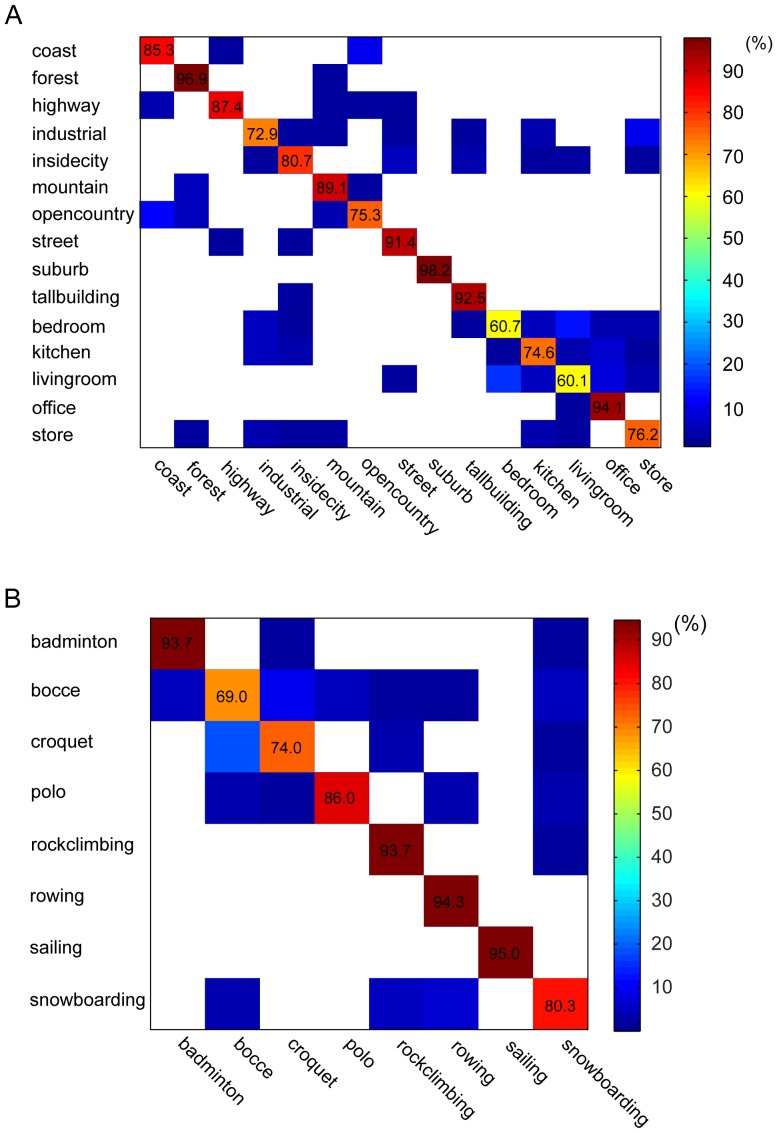
Confusion matrices. (A), Confusion matrix of the model performance on the dataset of 15 scenes. The average accuracy is 82.3%. (B), Confusion matrix of the model performance on the dataset of 8 sports. The average accuracy is 85.8%. In both A and B, the values at the empty matrix elements are 0.

Figure 10 shows the confusion matrices of the performance of our model on the two datasets. For the dataset of 15 scenes (Figure 10A), the mean error rates are 4.4% on the indoor scenes, 1.1% on the outdoor scenes, and 0.8% on the indoor vs. outdoor scenes, i.e., most of the misclassifications occurred within the indoor scenes. Our model achieved better performance on the outdoor scenes (87.0%) than on the indoor scenes (73.1%). In the worst cases, our model misclassified 16.3% of the living-room scenes as the bedroom scenes, and 11.7% of the open-country scenes as the coast scenes. On a subset of the dataset of 15 scenes, i.e., coast, forest, highway, inside city, mountain, open country, street, and tall building, which is another popular dataset of scene categories [8], our model achieved an accuracy of 87.3%, the same as the state-of-the-art result (87%) [48]. On the dataset of 8 sports (Figure 10B), the model misclassified 18.0% of the croquet scenes as the bocce category because these two scene categories are quite similar.

As a final note, the large numbers of the NSSs did not adversely affect the generalization power of our model for two reasons. First, as described above, we selected a set of NSSs with high information. Second, we compiled NSSs separately from each of the categories and built a master book of NSSs. The redundancy in the master book presumably limited over-fitting via the cross-validation procedure.

## Discussion

### Natural scene structures

In this study, we proposed NSSs as an intermediate-level representation of natural visual scenes. Each NSS is a concatenation of visual features in multi-scale circular patches in a hexagonal configuration. The NSSs encompass all possible concatenations of local features in natural scenes, including smooth patterns of luminance, textures, edges, junctions, and any combinations of these four patterns of luminance. The only limitations on the possible concatenations are induced by the clustering procedures, which can be made looser or tighter depending on specific applications. There are several advantages of the NSSs over usual local visual features. First, the NSSs provide a classification of natural scene patches for the purpose of generalization. Second, since the NSSs are topology-conserving, multi-scale, intermediate-level structures with sizes considerably larger than usual local features and encode local scaling-invariance, they can be very informative for specific applications and robust against noises and changes in scale. Third, there is no need to perform any image-based processing or to detection specific features or combinations of specific features. Fourth, spatial information in visual scenes can be assessed by examining the spatial arrangements of the NSSs at even larger scales. Finally, encoding of natural scenes in terms of the NSSs is more or less equivalent to specifying the spatial arrangements of the NSSs. It is worthy of pointing out that the NSSs proposed here share some similarity to the fragments advocated by several researchers [52], [53]. Because we provided a principled way to compile NSSs at multiple scales and to examine their spatial arrangements at larger scales, the NSSs proposed here, with all the above advantages, are a novel contribution and represent an addition to current research on natural scene statistics and their relationships to vision [54]–[58]. Also, as explained in detail in section “Relationship to other work”, the NSSs proposed here are different from other recent work on computational models of scene classification and models of neuronal responses to natural scenes.

We compiled NSSs from two widely used datasets of natural scenes, examined a range of statistics of the NSSs, and found that the NSSs contain a range of information about natural scenes. We also used adjacency matrices to model the spatial arrangements of the NSSs at larger scales. For scene classification, we selected a set of NSSs with high information and used the occurring frequencies of the NSSs and the eigenvalues of the adjacency matrices to classify the scenes in the datasets. We found that the classification performance of this model is significantly improved by concatenating visual features at multiple spatial locations (i.e., the hexagonal configuration) and scales, and is comparable to or better than the best current models on the two datasets. These results show that the NSSs proposed here are a useful intermediate-level representation of natural visual scenes.

### Possible neural representations of NSSs

The computational model of the NSSs and scene classification proposed here does not have any direct neurobiological support; nonetheless, it is useful to speculate how visual neurons can encode the NSSs. Along the ventral pathway of the primate visual system, neurons assemble progressively visual features. In V1, neurons are tuned to simple features such as oriented bars; neurons in V2 respond selectively to multiple simple features [28]; and in V4, neurons respond selectively to simple shapes [2], [30]. In the IT area, neurons respond selectively to complex features and the responses show tolerance to views, scales, positions, and poses [1]. Given this hierarchical coding strategy, it is plausible that populations of neurons along the ventral pathway encode the NSSs proposed here (see also Possible neural codes of natural scenes and Figure 1).

As explained in sections “Possible neural codes of natural scenes” and “Relationship to other work” (see also Figure 1 and Figure 3), the NSSs are ensembles of features in natural scenes obtained by three operations, i.e., categorization (via clustering), projection, and concatenation. These operations can be roughly mapped to nonlinear tuning, nonlinear filtering, and integrating multiple inputs by visual neurons, respectively. Thus, it is plausible that the neural circuitry and neuronal responses along the ventral pathway may implement these operations, as elaborated further below.

Each NSS has two layers (Figure 3). In the first layer, the basic visual features are ICs, similar to the features to which V1 neurons are tuned. The feature element *a_i_* of each circular patch is the root mean square of the amplitudes of the ICs in an IC cluster (Equation (1)). Although *a_i_* may not be mapped to the response of any single V1 neuron, it is a simple nonlinear function (as in the standard LN model of V1 neurons) of the responses of a small number of V1 neurons whose tuning to orientation, frequency, and phase are similar (since the ICs in an IC cluster have similar parameters). This neural model of *a_i_* would be a generalization of the standard LN model of V1 neurons to the population level [26].

In the second layer, each NSS in the space of the feature vectors is characterized by a set of dominant IC clusters at multiple spatial scales. As a result, each NSS is a function of the responses of a population of V1 neurons via linear and simple nonlinear operations (i.e., operations in the standard LN model of V1 neurons). This is also true for V2 neurons that are similar to V1 neurons, but only some of the NSSs can be functions of the responses of populations of V2 neurons that encode multiple orientations via linear and simple nonlinear operations. The rest of the NSSs are functions of the responses of these V2 neurons via complex nonlinear operations (i.e., more complex than the operations in the standard LN model) and the relative portion of these NSSs is unknown at this time. In the same fashion, some of the NSSs can be functions of the responses of populations of V4 and IT neurons via linear and simple nonlinear operations and some of the NSSs can be functions of the responses of populations of V4 and IT neurons via complex nonlinear operations. Thus, even though highly speculative, it is plausible that populations of neurons along the ventral pathway of the primate visual system encode the NSSs that include a full range of concatenations of visual features.

## Materials and Methods

We implemented our model in Matlab (Version 7.10.0.499) running on a Dell Optiplex 980 desktop (with an Intel Core i7 860 processor and 16G RAM).

### Datasets of natural scenes

We used two datasets of natural scene categories, the dataset of 15 scenes and the dataset of 8 sports [33], [59]. The first dataset contains 4,485 images of 10 categories of outdoor scenes (i.e., coast, forest, highway, industrial, inside city, mountain, open country, street, suburb, and tall building) and 5 categories of indoor scenes (i.e., bedroom, kitchen, living room, office, and store). Each category has 200 to 400 images of ∼ 300×250 pixels. Figure 2A shows sample images of the 15 scene categories.

The dataset of 8 sports contains 1,579 images of 8 scene categories of sports, i.e., badminton, bocce, croquet, polo, rock climbing, rowing, sailing, and snowboarding. Each category has 130 to 250 images of ∼ 900×1100 pixels, which were acquired at various camera distances. Figure 2B shows sample images of the 8 scene categories.

### Training and cross-validation

As in several other studies [33], [59], we randomly selected 100 samples of each category for training and used the rest of the images for testing for the dataset of 15 scenes; and for the dataset of 8 sports, we randomly selected 70 and 60 samples of each category for training and testing respectively. The classification accuracies reported in this paper are the averages of the accuracies obtained in 5 training-testing runs.

Cross-Validation (CV) is a way to control over-fitting and is widely used in pattern recognition and statistical modeling. We used five-fold CV on the training sets to select model parameters, which included: 1) the number of clusters in the K-means method, 2) *L_c_* and *N_c_*, the parameters for extracting spatial information, 3) *M_c_*, the threshold for selecting NSSs, and 4) the parameters of the SVM classifier. In this procedure, we separated the training data into five equal folds, tested the model on a single fold using the remaining 4 folds to train the model, and repeated this procedure on each of the five folds.

### Pre-processing

As in several other studies [33], [42], we sampled patches densely in the dataset of 15 scenes and the dataset of 8 sports. In dataset of 15 scenes, we sampled two sets of circular patches in hexagonal configurations at a step of 4 pixels. The diameters of the circular patches in the two sets were 16 and 24 pixels. In the dataset of 8 sports, we sampled four sets of circular patches in hexagonal configurations at a step of 8 pixels. The diameters of the circular patches in the four sets were 16, 24, 32, and 46 pixels. Since the sizes of images vary in the dataset of 8 sports, we resized the larger dimension to 840 pixels while maintaining the aspect ratios of the images.

### Testing on the dataset of 15 scenes

We randomly sampled 4×10^6^ patches at each spatial scale, performed ICA on the patches, and obtained 160 ICs that accounted for 99% of the variance of the patches. We fitted Gabor functions to the 160 ICs at each of the 2 scales, converted the fitted parameters into pre-set intervals (i.e., the scale of the Gaussian envelope to [0, +∞), the orientation of sinusoid carrier to [0, π), and the phase of the sinusoid carrier to [0, 2π)). We then classified the ICs of the patches at the two scales into 100 IC clusters using the K-means method with the Euclidean distance as a function of the parameters of the Gabor functions.

To compile the NSSs, for each scene category, we randomly selected 2×10^5^ patches, projected them to the IC clusters, and obtained 1,000 NSSs using the K-means method. The distance used in this step was the Euclidean distance between the root mean square of the amplitudes of the ICs in the IC clusters. We obtained 14,600 NSSs for this dataset.

To select NSSs for the SVM classifier, we set the threshold, *M_c_* = 7, the number of images in which the NSS occurred. We selected 11,028 NSSs, set *L_c_* = 3 and *N_c_* = 3, and used the 1- χ^2^ kernel in the SVM classifier (C  =  0.125).

### Testing on the dataset of 8 sports

We randomly sampled 4×10^6^ patches at each spatial scale, performed ICA on the patches, and obtained 160 ICs that accounted for 99% of the variance of the patches. We fitted Gabor functions to the ICs, converted the fitted parameters, and classified the ICs of the patches at 4 scales into 40 clusters using the K-means method with the Euclidean distance (see above). To compile NSSs, for each scene category, we randomly selected 3×10^5^ samples, projected them to the IC clusters, and obtained 600 NSSs using the K-means method (see above). We obtained 4,761 NSSs for this dataset. We used all the NSSs for classification, set *L_c_* = 5 and *N_c_* = 1, and used the 1- χ^2^ kernel in the SVM classifier (C  =  0.125).
